# Spatial suppression due to statistical regularities in a visual detection task

**DOI:** 10.3758/s13414-021-02330-0

**Published:** 2021-11-12

**Authors:** Dirk van Moorselaar, Jan Theeuwes

**Affiliations:** 1 Department of Experimental and Applied Psychology, Vrije Universiteit Amsterdam, Amsterdam, the Netherlands; 2 Institute of Brain and Behaviour Amsterdam, Amsterdam, the Netherlands

**Keywords:** Attention: space-based, Attentional capture, Visual search

## Abstract

Increasing evidence demonstrates that observers can learn the likely location of salient singleton distractors during visual search. To date, the reduced attentional capture at high-probability distractor locations has typically been examined using so called compound search, in which by design a target is always present. Here, we explored whether statistical distractor learning can also be observed in a visual detection task, in which participants respond target present if the singleton target is present and respond target absent when the singleton target is absent. If so, this allows us to examine suppression of the location that is likely to contain a distractor both in the presence, but critically also in the absence, of a priority signal generated by the target singleton. In an online variant of the additional singleton paradigm, observers had to indicate whether a unique shape was present or absent, while ignoring a colored singleton, which appeared with a higher probability in one specific location. We show that attentional capture was reduced, but not absent, at high-probability distractor locations, irrespective of whether the display contained a target or not. By contrast, target processing at the high-probability distractor location was selectively impaired on distractor-present displays. Moreover, all suppressive effects were characterized by a gradient such that suppression scaled with the distance to the high-probability distractor location. We conclude that statistical distractor learning can be examined in visual detection tasks, and discuss the implications for attentional suppression due to statistical learning.

A growing body of research indicates that whether visual information is attended, regardless whether it is relevant or irrelevant, is not only determined by the interaction between top-down and bottom-up processes but is also strongly influenced by previous selection episodes (Awh et al., 2012; Theeuwes, 2019; van Moorselaar & Slagter, 2020). Recent studies have demonstrated that regularities across trials not only influence what is more likely to be attended, but also what is more likely to be suppressed. For example, Wang and Theeuwes (2018c) showed that distractors were more efficiently ignored at locations that contained distractors with a higher probability. This effect demonstrates that people, often implicitly, can learn about spatial regularities of irrelevant items that must be ignored (Di Caro et al., 2019; Ferrante et al., 2018; van Moorselaar, Daneshtalab, & Slagter, 2020; Wang & Theeuwes, 2018b).

To account for reduced attentional capture at high-probability distractor locations, it was argued that statistical regularities cause passive lingering biases on a spatial priority map. Consequently, the high-probability distractor location competes less for attention relative to other locations. Consistent with spatially tuned suppression, (1) reduced distractor interference often scales with the distance from the distractor relative to the high-probability location (Wang & Theeuwes, 2018b, 2018c), and (2) target processing has been shown to be impaired at that location (Wang & Theeuwes, 2018c), although the latter effect appears specific to conditions where targets and distractors are defined within different dimensions and vary randomly (Zhang et al., 2019).

To date, statistical learning of distractor suppression has particularly been examined using the additional singleton task (Theeuwes, 1991, 1992) which is considered to be a “compound search” task. In compound search (Duncan, 1985; Theeuwes, 1992) participants search for one feature (e.g., diamond) and respond to another feature (e.g., orientation of element inside the target), which makes it possible to separate attentional selection from response selection (Theeuwes et al., 2006). Even though the advantages of such a paradigm have been plenty, it may be less optimal to examine attentional suppression because by design, in compound search a target is always present. If one wants to examine suppression stemming from statistical learning in the absence of a priority signal generated by target singletons, one has to employ a visual detection task in which observers decide whether target singletons are present or absent.

The most obvious advantage of visual detection (i.e., present/absent visual search) in the context of learned distractor suppression is that the effect of interest (i.e., distractors at high versus low-probability distractor locations) can be examined in the absence of a priority signal elicited by the target. This is especially useful when multiple high-probability locations are introduced within the same paradigm to, for example, investigate whether suppression can be feature-specific(Failing et al., 2019) or context-specific(Britton & Anderson, 2020). Moreover, target-absent displays allow for a clean reconstruction of spatial distractor gradients. Within-compound search, by design, on average, distractors further away from the high-probability location have a higher chance that the target is in close proximity to that location, and vice versa.

Even though this approach seems straightforward there is one important aspect that needs to be considered. In visual detection tasks involving pop-out targets, it is argued that present-absent decisions can be made without directing spatial attention to their location. Indeed, according to classic feature integration theory (Treisman & Gelade, 1980) and more recent versions (Müller et al., 2003), when participants have to detect singleton features, they can do so on the basis of pooled responses within so-called dimensional modules. Critically, while these modules signal the presence of unique information, they do so in a nonspatial manner, such that target presence can be determined without spatial attention. Similar notions have been put forward from a neurophysiological perspective. The idea is that attention is only required when ambiguities in neural coding need to be resolved (Luck & Ford, 1998)—for example, when multiple objects are presented within the same receptive field of neurons within the ventral object recognition pathway. In such a scenario, focal attention is needed to resolve the ambiguity and determine the correct response (Luck et al., 1997). If there are no ambiguities in the visual field, spatial attention is not needed to generate a present–absent response in visual detection (but see Theeuwes et al., 1999; Theeuwes et al., 2008).

In the present study we examined whether location-probability learning is also evident during present/absent search, and if so, whether it is characterized by a spatial gradient and impaired target processing at that location. For this purpose, in an online variant of the additional singleton paradigm (Theeuwes, 1992), participants had to indicate whether a search display contained a unique shape (i.e., the target), while, on distractor-present trials, ignoring a singleton distractor (see Fig. 1). Critically, the singleton distractor appeared with higher probability at one specific location. This allowed us to test whether participants can learn spatial regularities in a task that is hypothesized to not necessarily need spatial attention to generate a response.

**Fig. 1:**
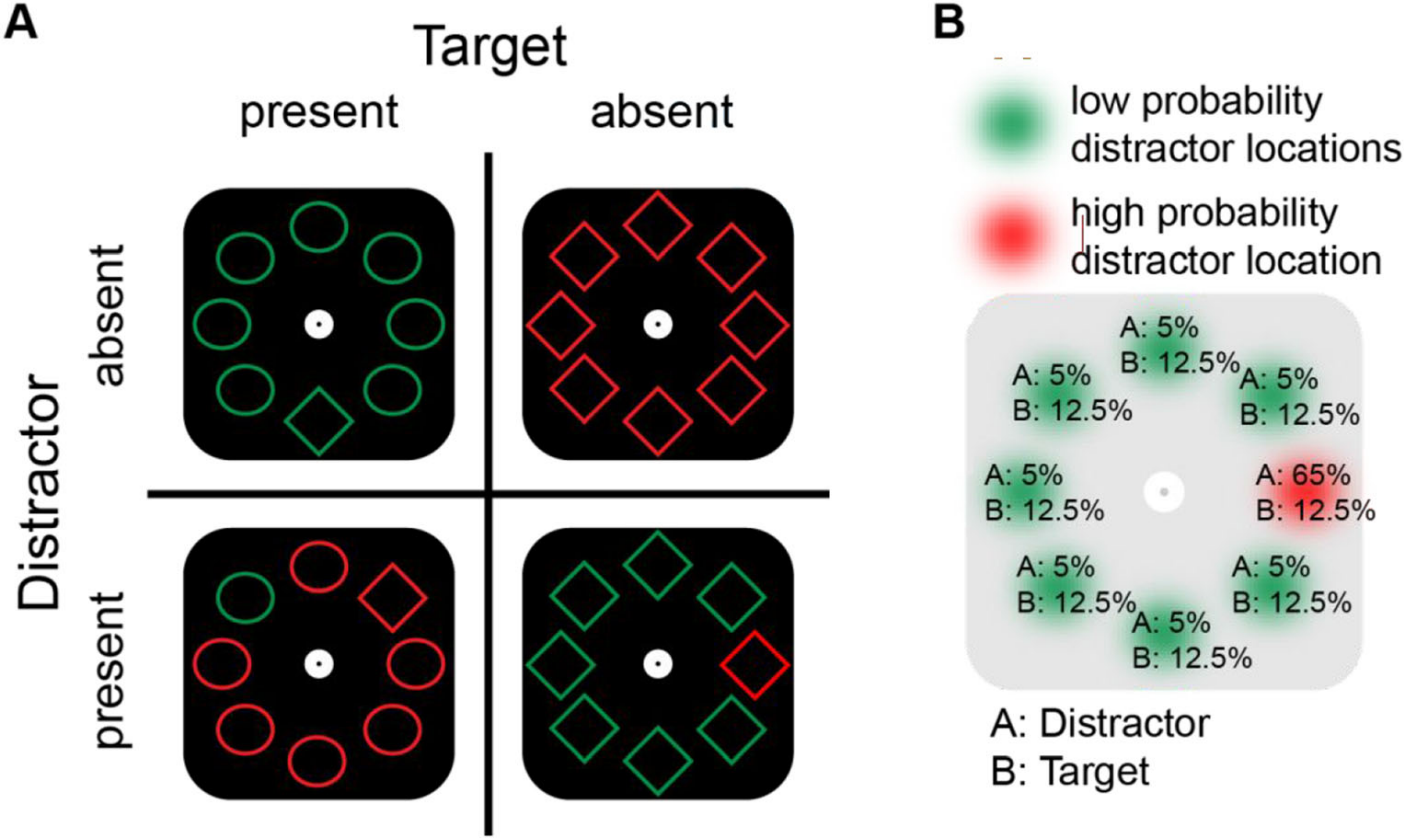
Experimental paradigm. **a** Example search display configurations. Per display, participants had to indicate whether a unique shape singleton was present or absent. The singleton distractor color was more likely to appear in one position along the imaginary circle. **b** Schematic representation of the spatial regularities of the distractor. Percentages at each location represent the probabilities of the distractor and the target, when present (i.e., half of the trials), appearing at a given location. (Color figure online)

## Methods

### Participants

The final sample contained 48 first year students (*M*_age_ = 21.6 years, range: 18–52 years; 41 females; descriptives of one participant was missing), which participated for research credits. No participants with a complete data set were excluded and sample size was justified with a power analysis based on the main effect of distractor location as reported in (Wang & Theeuwes, 2018c). With ${n}_p^2$ = 0.85 and *α* = 0.05, power for the critical test was >.99. The ethical committee of the Faculty of Behavioral and Movement sciences Vrije Universiteit approved the study, which was conformed to the Declaration of Helsinki and participants provided digital informed consent via Qualtrics (Qualtrics, Provo, UT), prior to participation.

### Task, stimuli, and procedure

As the experiment was conducted online, and we thus had no control over the experimental setting, for replication purposes we report pixels and RGB values to describe the stimuli. The experiment was created in OpenSesame v3 (Mathôt et al., 2012) using OSWEB (Version 1.3.11) and run using JATOS (Lange et al., 2015).

Each trial started with a 500-ms fixation display, that consisted of a white circle on a black background. Subsequently a search display appeared with eight equidistant shapes in a circular configuration around fixation (radius: 224 pixels), which remained visible for 2,000 ms or until response (see Fig. 1). Each display contained eight circles (radius: 45 pixels) or diamonds (100 × 100 pixels), each with a red (255/0/0) or a green (0/146/69) outline on a black background. On target-present trials (50%) one of the circles was replaced by a diamond of the same color, or vice versa. On distractor-present trials (50%) the outline of one of the nontarget shapes had a different color than the other stimuli in the display. Critically, while the target appeared with equal probability across all locations in the display, both in distractor-present and distractor-absent trials, the singleton distractor appeared with a higher probability (65%) at one of the eight locations (counterbalanced across participants^1^). In case of an incorrect or missing response the fixation circle turned red for 500-ms, whereas it remained white for 250-ms in case of a correct response.

Participants were instructed to keep their eyes at fixation, and to indicate via key press whether the target was present (press ‘p’) or absent (press ‘a’) while ignoring the singleton distractors. Participants, who were encouraged to respond as fast as possible, while keeping the number of errors to a minimum, completed seven blocks of 80 trials each (trial order randomized), preceded by a series of 15 practice trials. The practice block continued to repeat until average reaction time (RT) was below 1,500-ms and average accuracy was above 66%. Halfway through each block participants were given the opportunity for a short break, and at the end of each block they received feedback on their performance (i.e., mean RT and accuracy), while they were encouraged to take a break. After the last block, participants were first asked whether they noticed that one location had a higher distractor probability. Subsequently, a display with white circles, each with a unique identifier, corresponding to one of the search locations was shown and participants had to indicate (and if necessary, guess) which location they believed contained the singleton distractor most frequently throughout the experiment.

### Statistics

Search times analyses were limited to data of correct trials only. RTs were filtered in a two-step trimming procedure: trials with RTs shorter than 200 ms were excluded, after which data were trimmed on the basis of a cutoff value of 2.5 standard deviations from the mean per participant. Exclusion of incorrect responses (7.3%) and data trimming (3.1%) resulted in an overall loss of 10.4% of trials. Remaining RTs were analyzed with repeated-measures analyses of variance (ANOVAs), where reported *p* values are Greenhouse–Geiser corrected in case of sphericity violations, followed by planned comparisons with paired *t* tests using JASP software *(*JASP Team, 2018).

## Results and discussion

### Distractor interference

To assess whether distractors interfered with visual detection we conducted a repeated-measures ANOVA (RM-ANOVA), with within subject factors target presence (present, absent) and distractor presence (present, absent). Thus, unlike in compound search, in which a target is always present, we analyzed distractor interference as a function of whether the target was also present in the display (i.e., respond target is present), or whether the distractor was the only singleton in the display (i.e., respond target is absent). This analysis showed overall faster RTs in target absent than target present trials, target presence: *F*(1, 47) = 35.3, *p* < .001, ${n}_p^2$ = 0.43, and reliable distractor interference, distractor presence: *F*(1, 47) = 94.2, *p* < .001, ${n}_p^2$ = 0.67, which was most pronounced in target absent displays, interaction: *F*(1, 47) = 49.6, *p* < .001, ${n}_p^2$ = 0.51.

**Fig. 2:**
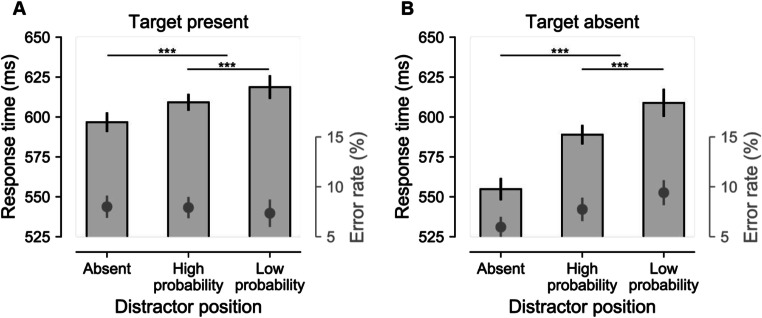
Reduced, but not absent distractor interference at high-probability distractor locations. Response times are visualized by bars (left *y*-axis) and error rate is visualized by grey insets (right *y*-axis). RTs and error rates as a function of distractor location in target-present (**a**) and target-absent (**b**) displays. All error bars here and in subsequent plots represent 95% within-subject confidence intervals (Morey, 2008).

Next, we entered RTs into a RM-ANOVA with within subjects’ factors target presence (present, absent) and distractor location (high-probability distractor location, low-probability distractor location) to analyze whether distractor interference was modulated by the uneven distribution of distractor locations. In doing so, we made sure that trials with targets at the high-probability location were excluded, so that reported effects were not inflated by impaired target processing at the location where we expected suppression to be most pronounced (van Moorselaar, Lampers, et al., 2020). As visualized in Figure 2, and reflected by a main effect of Distractor location (*F* (1, 47) = 21.9, *p* < 0.001, ${n}_p^2$ = 0.32), distractors were more efficiently ignored at high relative to low-probability locations, both in displays with, *t*(47) = 2.6, *p* = .011, *d* = 0.38, and without a target, *t*(47) = 4.5, *p* < .001, *d* = 0.64. Also, a trending interaction, *F*(1, 47) = 3.6, *p* = .064, ${n}_p^2$ = 0.071, suggested that the benefit at the high-probability location was more pronounced in target absent displays. Nevertheless, at high-probability distractor locations distractors continued to reliable interfere with response relative to distractor-absent displays, target present: *t*(47) = 4.4, *p* < .001, *d* = 0.63; target absent: *t*(47) = 10.5, *p* < .001, *d* = 1.52. The same analysis on error rate yielded a reliable interaction, *F*(1, 47) = 7.2, *p* = .01, ${n}_p^2$ = 0.13, showing lower error rate at the high relative to the low-probability location in target absent displays, *t*(47) = 2.8, *p* = .007, *d* = 0.41, whereas there was no such difference in target present displays, *t*(47) = 1.0, *p* = .31, *d* = 0.15, excluding an alternative explanation in terms of a speed–accuracy trade-off. Together, these findings show that learned distractor suppression is not exclusive to compound search tasks, but can also be observed in visual detection paradigms.

### Intertrial (location) priming

To exclude the possibility that the observed suppression does not reflect statistical learning of location probabilities, but instead is completely driven by intertrial priming (Maljkovic & Nakayama, 1994), the typical procedure is to repeat the main analysis after excluding all distractor location repetitions. Here, we applied the same approach, and in addition controlled for response priming by also excluding all trials where the response was the same as on the previous trial. This analysis mimicked the main findings, although there no longer was an apparent difference between target present and absent displays, *F*(1, 47) = 1.0, *p* = .30, ${n}_p^2$ = 0.023; *BF*_*01*_ = 11.9. Yet critically distractors were more efficiently ignored at high-probability distractor locations, *F*(1, 47) = 4.9, *p* = .032, ${n}_p^2$ = 0.095. Note, however, that a more refined priming control should not be limited to intertrial distractor location repetitions and in this case response priming, but include all transitions that potentially modulate statistical location learning (Zhang et al., 2019). Excluding all such transitions (e.g., D_n-1_– D_n_, D_n-1_– T_n_, T_n-1_– D_n_, T_n-1_– T_n_), however, markedly reduces the number of observations and thus a method, where data is not averaged but instead grouped per participant and hence allows for the inclusion of control variables, is arguably a better control to validate the statistical learning effect above and beyond intertrial priming (van Moorselaar, Daneshtalab, & Slagter, 2020). We therefore also ran a linear mixed model (Bates et al., 2014; for details, see Table 1 note) with various forms of intertrial priming (i.e., display, location, feature) as a control. This analysis showed that while various forms of intertrial priming contributed to the observed difference between high and low-probability distractor locations (see significant priming effects in Table 1), overall RTs remained reliably faster for distractors at high relative to low-probability locations when these forms of priming were taken into account, *t*(60) = 4.2, *p* < .001 (see Table 1).

**Table 1:** Estimates for mixed-effects model, using Satterthwaite’s method for approximating degrees of freedom (Luke, 2017)

		*β*	*SE*	*df*	*t*	*p*
Distractor location	High vs. low probability***	−14.3	3.4	60	4.2	<.001
Display priming	D_n-1_– D_n_	3.7	4.0	11878	0.9	.35
	T_n-1_– T_n_***	17.2	4.9	11861	3.5	<.001
Location priming	D_n-1_– D_n_	−4.7	4.0	11858	1.2	.24
	D_n-1_– T_n_**	26.5	8.8	11874	3.0	<.01
	T_n-1_– D_n_	8.8	5.3	11875	1.7	.10
	T_n-1_– T_n_**	−26.4	8.2	11868	3.2	<.01
Shape priming	D_n-1_– D_n_***	−15.0	3.8	11877	4.0	<.001
	T_n-1_– T_n_	−10.2	5.4	11849	1.9	.060
Color priming	D_n-1_– D_n_	1.6	3.8	11870	0.4	.67
	T_n-1_– T_n_	−6.0	5.4	11872	1.1	.27

*Note*. The model had a participant grouping variable, with a random effect structure including an intercept and distractor location—levels: high-probability, low-probability, absent; contrast (1,−1,0) as fixed variables various forms of intertrial display priming (D_n-1 present_– D_n present_, T_n-1 present_– T_n present_), location priming (D_n-1_– D_n_, D_n-1_– T_n_, T_n-1_– D_n_, T_n-1_– T_n_) and feature priming (D_n-1 color_– D_n color_, D_n-1 shape_– D_n shape_ , T_n-1 color_– T_n color,_ D_n-1 shape_– D_n shape_). The table shows the unstandardized estimates (*β*), the standard error (*SE*), estimated degrees of freedom (*df*) and the corresponding t and p values. We used sum coding (−1 vs. 1) for all control factors in the model (−1 = no priming, 1 = priming)

### Impaired target processing

**Fig. 3:**
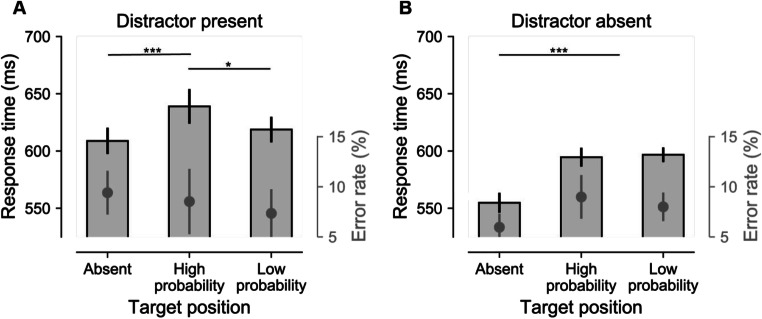
Impaired target processing at high-probability distractor locations, but only in distractor-present displays. Response times are visualized by bars (left *y*-axis) and error rate is visualized by grey insets (right *y*-axis). RTs and error rates as a function of target location in distractor-present (**a**) and distractor-absent (**b**) displays

In many studies in which the distractor features vary randomly across trials (as was the case here), the reduced distractor interference at the high-probability distractor location is accompanied by impaired target processing at that location (Wang & Theeuwes, 2018c; Wang, van Driel, et al., 2019). To investigate whether such feature-blind suppression was also evident here, RTs were analyzed with a RM-ANOVA with within-subjects’ factors distractor presence (present, absent) and target location (high-probability distractor location, low-probability distractor location). Trials with distractors at the high-probability distractor location were excluded so that any differences could not simply be explained by reduced distractor interference at that location. As visualized in Fig. 3, and reflected by an interaction, *F*(1, 47) = 5.4, *p* = .024, ${n}_p^2$ = 0.10, target processing was impaired at the high-probability location in distractor-present displays, *t*(47) = 2.4, *p* = .02, *d* = 0.35, but not in distractor-absent displays, *t*(47) = 0.6, *p* = .57, *d* = 0.083; *BF*_*01*_ = 5.4. The same analysis on error rates yielded no significant effects (all *F*s < 1.2, all *p*s > .27). These findings show that when two singletons were present, generating ambiguity in the display, target processing at the high-probability location was impaired, consistent with the notion that this location is suppressed. However, in the absence of a distractor, target processing was unaffected by spatial position.

### Spatial distribution of suppression

**Fig. 4:**
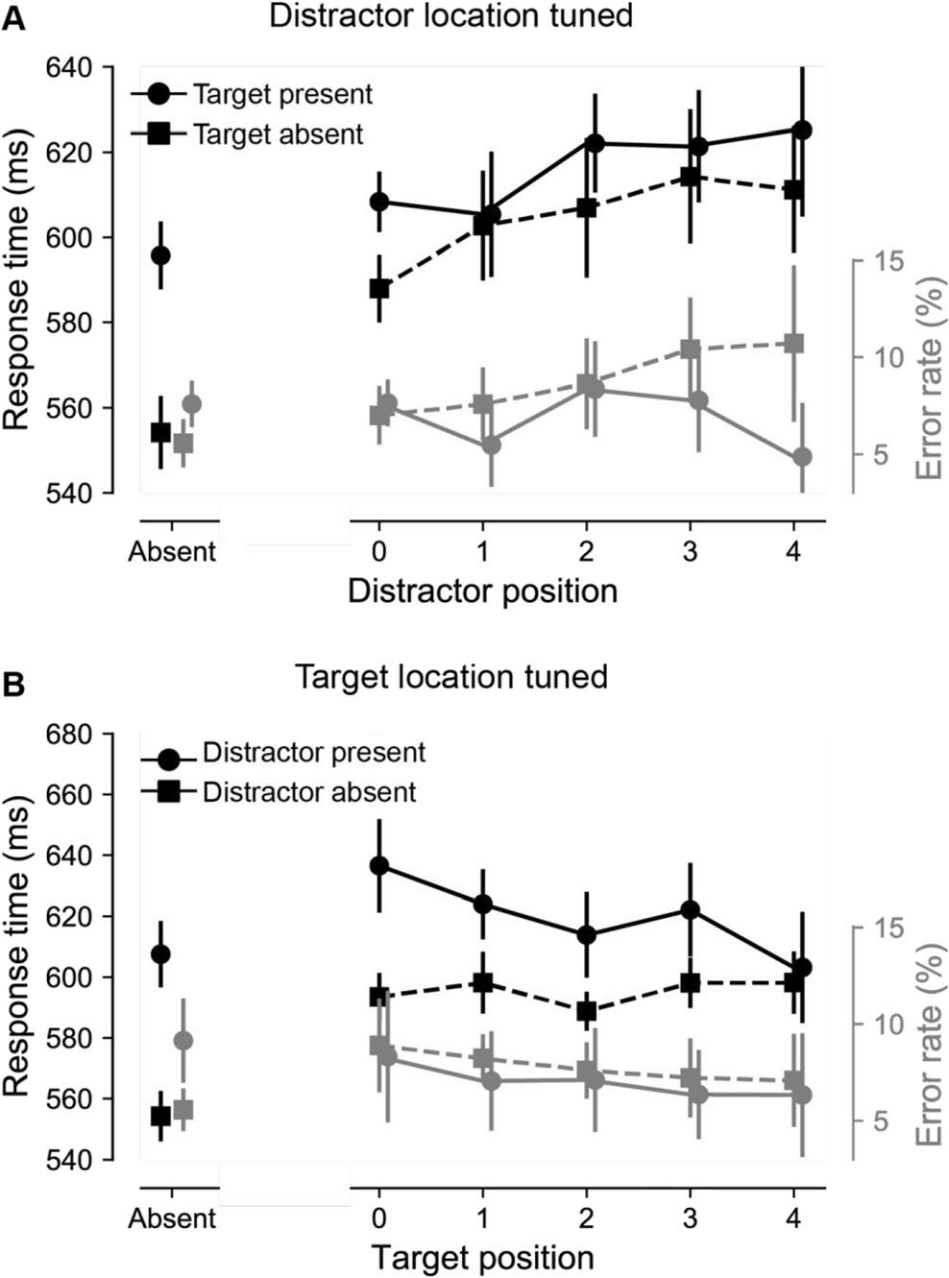
Gradients around the high-probability distractor location as a function of distractor (top) or target position (bottom). **a** Mean RT (black; left *y*-axis) and error rate (grey; right *y*-axis) for target-present responses (circle markers) and target-absent responses (square markers) for both distractor-absent trials (left on the *x*-axis) as well as by distance from the distractor to the high-probability distractor location (labels 0–4 on the *x*-axis). **b** Mean RT (black; left *y*-axis) and error rate (grey; right *y*-axis) for target absent responses and target present responses as a function of distance (0–4) from the target to the high-probability distractor location, separately for distractor-absent (square markers) and distractor-present (circle markers) displays. Note that in target distractor tuned analyses (**a**) targets at high-probability distractor locations were excluded, whereas in target tuned analyses, (**b**) distractors at high-probability distractor locations were excluded such that matching data points marked by circles in **a** and **b** do not perfectly overlap

To further characterize the observed effects, we examined how suppression changed as a function of the distance relative to the high-probability location. For this purpose, data used in distractor and target tuned analyses were binned as a function of the number of positions between the stimulus location and the high-probability location such that at distance 0, the stimulus of interest appeared at the high-probability location, at distance 1, it appeared next to that location, and so on. A distractor tuned RM-ANOVA with within subjects’ factors target presence (present, absent) and distance (0–4 bins) yielded a main effect of distance, *F*(3, 150) = 3.7, *p* = .012, ${n}_p^2$ = 0.072, which was characterized by a linear trend, *t*(188) = 3.6, *p* < .001 (see Fig. 4a). Note, however, that the effect of distance was no longer significant when position 0 was excluded, *F*(3, 141) = 1.7, *p* = .18; target-present*BF*_*01*_ = 5.9; target-absent*BF*_*01*_ = 20.4. The same analysis on error rate showed no main effect of distance nor an interaction (all *F*s < 2.3, all *p*s > .06).

In line with target processing being selectively impaired in distractor-present displays, the target tuned analysis yielded a reliable interaction, *F*(4, 188) = 3.0, *p* = .02, ${n}_p^2$ = 0.06, reflecting a spatial gradient in distractor-present, *F*(3, 150) = 3.2, *p* = .022, ${n}_p^2$ = 0.064, exhibiting a linear trend, *t*(188) = 3.1, *p* = .002, but not in distractor-absent displays (*F* (3, 151) = 1.1, *p* = 0.34, ${n}_p^2$ = 0.023). Again, the effect of Distance in distractor-present displays was no longer significant when position 0 was excluded, *F*(2, 116) = 1.9, *p* = .14, ${n}_p^2$ = 0.039 (see Fig. 4b). The same analysis on error rate showed no effects (all *F*s < 1.0, all *p*s > .33).

### Awareness of the high-probability distractor location

Finally, we examined whether participants noticed that distractors appeared with higher probability at a given location. Unlike in compound search, in which statistical regularities often remain implicit (Wang & Theeuwes, 2018c), the current paradigm also contained target absent trials, which may have made the spatial distractor imbalance more apparent. However, out of 40 participants that completed the implicit learning questionnaire, 13 indicated the correct location, of which only five indicated that they actually noticed the spatial imbalance, suggesting that observed effects do not reflect a deliberate strategy, but instead, at least in most participants, implicit statistical learning.

## General discussion

The present study investigated whether learned distractor suppression is also evident in a target detection task in which participants decide whether target singletons are present or absent. Previous studies using compound search, in which participants respond to a feature of the target (e.g., line orientation of element inside), have shown that distractors presented at high-probability distractor locations are suppressed relatively to all other location (Ferrante et al., 2018; Goschy et al., 2014; Leber et al., 2016; Wang & Theeuwes, 2018a, 2018c). Here, we show that such suppression is also observed using visual detection tasks. Specifically, attentional capture by salient distractors was reduced, both when the distractor was the only singleton in the display, and when it was accompanied by a target singleton. Moreover, target detection was impaired when targets happened to be presented at the high-probability distractor location, although this effect was not observed when the target was the only singleton present in the display. Also, suppression was characterized by a spatial gradient, with suppression gradually decaying as a function of the relative distance to the high-probability location.

The observed distractor suppression at the high-probability location was characterized by a spatial gradient, both in displays with and without a target singleton. These findings suggest that distractor suppression occurs even in a task that does not necessarily require spatial attention to resolve. Indeed, it has been argued that in feature detection tasks, unique activity in the relevant feature map should allow to generate a target present response without the need to direct attention to the feature’s singleton location (Müller et al., 2003; Treisman, 1988). Yet the current study shows that spatial attention does play a large role in these kind of detection tasks as we observed a clear spatial gradient of suppression.

The data allow us to interpret these findings in more detail. In target-absent trials, participants should be able to respond “target absent” because of the absence of activity in the relevant (target) feature map (Treisman, 1988). Yet we do see a clear gradient (see Fig. 4a, dotted lines) such that generating this “target-absent” response is faster when the distractor is at the high-probability location and the effect scales with distance from this location. This implies that the distractor generates (preattentive) feature activity and that this needs to be checked to determine whether this feature activity stems from the presence of a target or the presence of a distractor singleton. The results elegantly show that participants are faster to decide “not the target but a distractor” when distractors are presented at the high-probability location, and this decision scales with distance for this location. In this sense, statistical regularities generate a “scaled with distance” decision bias. These finding are comparable to eye movement studies examining the speed with which observers can disengage their eyes from a high-probability location. These studies not only showed fewer saccades directed to the high-probability location (suggesting suppression), but also that participants were faster to move their eyes away from the distractor when it was presented at a high relatively to a low-probability location. Also, here, the decision “this is not the target but a distractor” was sped up for locations that often contained a distractor (Sauter et al., 2020; Wang, Samara, & Theeuwes, 2019).

When examining the target location tuned analysis the results are somewhat different. In target displays in which a distractor was also present, there was a clear target gradient, with slowest responses for targets at the high-probability location. This suggests proactive suppression such that regardless of whether a target or a distractor is presented at that location, the location is suppressed. As noted above, this proactive suppression at the spatial priority map results in faster responses when a distractor is presented there, but higher costs when a target is presented there. However, when the target was not accompanied by a distractor, there was no longer an effect of target position, suggesting that in those conditions participants generate a response without consulting the spatial priority map. This latter finding is consistent with theories assuming that visual detection can rely on dimensional modules that signal the presence of a feature singleton, without the need for spatial attention (Chan & Hayward, 2009; Kumada, 1999; Müller et al., 2003; Treisman & Gelade, 1980). In this respect, it should be noted that responses to displays containing only a target were reliably faster compared to displays that only contained a distractor.^2^ One explanation for the observed discrepancy is that the target dimension is given priority such that saliency signals within this dimension module were sufficient to elicit a target present response, as long as that signal was not in competition with another more salient singleton. As soon as there is competition of a signal from another dimension, further processing of the input in the master saliency map is required in order to eliminate that the saliency signal is from the potential target (Müller et al., 1995). In other words, in visual detection tasks displays containing a distractor are ambiguous, either because two singletons are processed simultaneously and therefore compete for representation (Desimone & Duncan, 1995; Luck & Ford, 1998) or because a check is needed to determine the source of the priority signal This implies that we cannot conclude that the high-probability location was not suppressed; we can only conclude that the spatial priority map was not checked when for when generating a target present response.

The current results indicate that in a target detection task, the location that is most likely to contain a distractor is suppressed relative to all locations. Even in a task that does not necessarily requires spatial attention, the spatial regularities are learned and are used to optimize performance. Similar to what has been concluded with compound search task, we assume that learning affects the weights within the priority map such that a location that is more likely to contain a distractor becomes proactively suppressed (Ferrante et al., 2018; Wang & Theeuwes, 2018a; Wang & Theeuwes, 2018c; Wang, van Driel, et al., 2019)

As a final note, the current task is very well suited for neurophysiological studies examining learned distractor inhibition. Note that suppression was also evident in target absent displays, making it is possible to measure the “pure” suppressive response to distractors without a bias from other salient signal present in the display (Desimone & Duncan, 1995). Moreover, it is even possible to examine the suppression of the high-probability location in displays in which there are no salient singletons whatsoever. This approach holds a big promise for future studies.

## Footnotes

1 Due to a counterbalancing mistake, one location (i.e., bottom left) contained five observations, whereas another location (i.e., left) contained seven observations instead of six.

2 This explorative comparison was done after excluding trials with distractors or targets at high-probability distractor locations, *t*(47) = 2.3, *p* = .029.
